# Transcription-Associated Mutagenesis Increases Protein Sequence Diversity More Effectively than Does Random Mutagenesis in *Escherichia coli*

**DOI:** 10.1371/journal.pone.0010567

**Published:** 2010-05-10

**Authors:** Hyunchul Kim, Baek-Seok Lee, Masaru Tomita, Akio Kanai

**Affiliations:** 1 Institute for Advanced Biosciences, Keio University, Tsuruoka, Japan; 2 Systems Biology Program, Graduate School of Media and Governance, Keio University, Fujisawa, Japan; Cairo University, Egypt

## Abstract

**Background:**

During transcription, the nontranscribed DNA strand becomes single-stranded DNA (ssDNA), which can form secondary structures. Unpaired bases in the ssDNA are less protected from mutagens and hence experience more mutations than do paired bases. These mutations are called transcription-associated mutations. Transcription-associated mutagenesis is increased under stress and depends on the DNA sequence. Therefore, selection might significantly influence protein-coding sequences in terms of the transcription-associated mutability per transcription event under stress to improve the survival of *Escherichia coli*.

**Methodology/Principal Findings:**

The mutability index (*MI*) was developed by Wright *et al.* to estimate the relative transcription-associated mutability of bases per transcription event. Using the most stable fold of each ssDNA that have an average length *n*, *MI* was defined as (the number of folds in which the base is unpaired)/*n*×(highest –ΔG of all *n* folds in which the base is unpaired), where ΔG is the free energy. The *MI* values show a significant correlation with mutation data under stress but not with spontaneous mutations in *E. coli*. Protein sequence diversity is preferred under stress but not under favorable conditions. Therefore, we evaluated the selection pressure on *MI* in terms of the protein sequence diversity for all the protein-coding sequences in *E. coli*. The distributions of the *MI* values were lower at bases that could be substituted with each of the other three bases without affecting the amino acid sequence than at bases that could not be so substituted. Start codons had lower distributions of *MI* values than did nonstart codons.

**Conclusions/Significance:**

Our results suggest that the majority of protein-coding sequences have evolved to promote protein sequence diversity and to reduce gene knockout under stress. Consequently, transcription-associated mutagenesis increases protein sequence diversity more effectively than does random mutagenesis under stress. Nonrandom transcription-associated mutagenesis under stress should improve the survival of *E. coli*.

## Introduction

During transcription, the nontranscribed strand becomes single stranded, whereas the transcribed strand forms a complex with RNA polymerase and the nascent RNA transcript [1]. The ssDNA is much more vulnerable to most mutagens than is the double-stranded DNA [2] because it is not protected by pairing [3]. The resulting mutations include single-base substitutions [2], [4], [5], [6] and insertions/deletions (indels) [4], [7] and are called transcription-associated mutations [8] or transcription-induced mutations [9]. Therefore, transcription-associated mutagenesis should be active on the nontranscribed strands, in highly transcribed DNA regions, and in cells under stress where high levels of mutagens are active. The existence and significance of transcription-associated mutagenesis is widely supported. For example, increased mutations have been observed in highly transcribed regions in diverse species, such as *Escherichia coli* [9], [10], [11], yeast [8], and humans [12], [13]. The nontranscribed strand is thought to have greater numbers of mutations than the transcribed strand in *E. coli* [6], [9], [11] and humans [14], [15], [16]. The larger numbers of mutations on the nontranscribed strand are partly but not solely attributable to the activity of the transcription-coupled DNA repair system, which acts on the transcribed strand, in *E. coli* [6]. Transcription-associated mutations are considered to occur regardless of specific secondary structures [17]. Transcription-associated mutagenesis becomes active under stress [18], and occurs in both genomic DNA and plasmid DNA in *E. coli* [17]. Therefore, transcription-associated mutagenesis is considered to be an intrinsic source of mutations [5], [8], [11], [19]. Furthermore, transcription-associated mutations occur at a level that affects the genomic composition of T7 bacteriophage [20]. As transcription-associated mutations occurs asymmetrically on the transcribed and nontranscribed strands [6], when all the transcription-associated mutations that occur in a cell are not repaired, its two daughter cells have different genomic DNA. Transcription-coupled DNA repair act selectively on the transcribed strand [21]. Most, if not all, mutations on nontranscribed strands would be expressed only after replication. Therefore, transcription-associated mutations should predominantly exert their effects not on the transcribing cells but on their descendant cells. The mutation rate of *E. coli* is lower than one base pair per genome per replication [22]. Therefore, *E. coli* cells would often contain one transcription-associated mutation and no other types of mutations in a genomic strand and no mutations in the other genomic strand until cell division when transcription-associated mutagenesis operates. In such cases, the unmutated genomic DNA strand of the cell is inherited by one of its two daughter cells. Consequently, transcription-associated mutagenesis can be considered a safe way for dividing cells to rapidly increase the sequence diversity of the next generation.

Transcription-associated mutagenesis has often been investigated in reversion assays under stress [6], [23]. The nonrevertants in the reversion assays are often assumed to be nondividing cells. In reversion assays, organisms such as *E. coli* are engineered to divide actively and hence form large detectable viable colonies only when one or more of the requisite mutations occurs. *E. coli* can continue to divide slowly by living on the debris of other cells [24]. For example, *E. coli* can survive in batch cultures without any addition of nutrients for many months [25]. Therefore, the possibility that such nonrevertants divide as many times as they die cannot be excluded. If cells are assumed to be nondividing, it is difficult to see how *E. coli* could survive the high mutation rate experienced by revertants. However, such a high mutation rate can be explained if the *E. coli* cells live on cell debris and if transcription-associated mutagenesis plays a significant role. We describe here a possible scenario. After the nonrevertants are plated, the *E. coli* cells continue to divide slowly by living on the debris of other cells, perhaps following the death phase, in which about 99% of cells die. When the *E. coli* cells experience stress, transcription-associated mutagenesis increases [18]. *E. coli* safely increases its sequence diversity in the daughter cells whenever all the transcription-associated mutations that occur are not repaired correctly before cell division. Whenever detrimental mutations occur alone or in combination with preexisting mutations, the cells die and produce cell debris. The surviving cells gradually accumulate mutations as cell division recurs. While mutation and selection occur, transcription-associated mutations can occur at the sites that produce revertants. Therefore, the multiple mutations of the revertants can be interpreted as the result of mutation and selection through many generations, which would significantly reduce the estimated mutation rate. Revertants are usually counted 48 h after plating [6], [23], which is longer than the overnight or one day incubation typically required for colonies to appear when nonrevertant *E. coli* cells are plated on rich medium. This late appearance of revertants can be attributed to slow cell division, a relatively low mutation rate, and limited numbers of surviving cells per generation. The mismatched DNA base pairing caused by mutations can result in cross-strand deamination *in vitro* [26], [27], so it is possible for transcription-associated mutagenesis to rescue nondividing cells.

The ssDNA of the nontranscribed strand forms secondary structures [18], which have different stability. Therefore, some ssDNA sequences are sustained for a longer time than others. Consequently, individual bases in the nontranscribed strand display different transcription-associated mutability per transcription event, depending on the period during which the base is unpaired [28]. Transcription-associated mutability per transcription event has been estimated by a few methods [3], [28]. Among them (see “Materials and Methods” for details), the mutability index (*MI*), developed by Wright *et al.*, focuses on transcription-associated mutagenesis under stress conditions [28]. The following is a description of the method of Wright *et al.* [28]. Because the length of ssDNA can vary, the length of the ssDNA was simplified to an average value *n* [28]. Therefore, any given base is assumed to belong to *n* ssDNA. The most stable fold of each ssDNA was identified, together with its –ΔG (the negative free energy) and pairing information. Using these *n* folds containing a given base, the *MI* of the given base was defined as (number of folds in which the base is unpaired)/*n*×(highest −ΔG of all *n* folds in which the base is unpaired). After scanning the average ssDNA length (*n*) for high −ΔG to best match known *in vivo* hotspot data for *E. coli*, Wright *et al.* set the average length of ssDNA (*n*) to 30 nt. The calculated *MI* values showed a positive significant correlation with the *in vivo* mutation data from reversion assays, but not with spontaneous mutation data [28]. This result suggests that *MI* can represent the relative transcription-associated mutability per transcription event in *E. coli* under the conditions of the reversion assays. Nontranscription-associated mutations were not excluded from either of the mutation data sets used for the validation tests. Therefore, the validation results imply that transcription-associated mutations constitute a large fraction of the total mutations in highly transcribed regions under the conditions of the reversion assays. Conversely, the invalidation of *MI* by the spontaneous mutation data might be attributable to either or both of the following two causes. First, transcription-associated mutations might not constitute a large enough fraction of the total spontaneous mutations to show a correlation. Second, the *MI* for the 30-nt ssDNA might not represent transcription-associated mutability per transcription event well under favorable conditions. This may occur because the average length of ssDNA was set to 30 nt by screening hotspot data that were obtained under stress [28].

Transcription-associated mutability per transcription event depends on the secondary structures formed by the DNA sequence [23], [28], [29] and can therefore be influenced by selection [3]. Every mutation does not exert the same effect. In protein-coding sequences, silent mutations circumvent potentially deleterious effects but do not increase the protein sequence diversity. Protein sequence diversity is advantageous under stress but not under conditions of spontaneous mutation. Interestingly, *MI* showed a positive significant correlation with mutation data under stress but not under conditions of spontaneous mutation [28]. Transcription-associated mutagenesis has been strongly suggested to play important roles under stress. For example, transcription-associated mutations are abundant under stress [18], [28] and in highly transcribed regions [10], [11]. Transcribed regions under a given stress might be better targets for beneficial mutations under that stress [30]. The *MI* validation by Wright *et al*. [28] suggested that transcription-associated mutagenesis is responsible for a large fraction of total mutations. Therefore, protein-coding sequences might have evolved to effectively increase protein sequence diversity by controlling transcription-associated mutability per transcription event under stress and hence *MI* values. If nonrandom *MI* values have been shaped within protein-coding sequences, it would provide a clear advantage for the survival of *E. coli*. In the present study, we analyzed the effect of selection on *MI* values using 4,132 protein-coding sequences from *E. coli* K12 MG1655, a fully sequenced *E. coli* strain. Our results show that bases have higher *MI* values when one or more of the three possible single-base substitutions at that base changes the encoded amino acid than when none of the three single-base substitutions at the base changes the encoded amino acid. Start codons have evolved to have lower *MI* values than nonstart codons. The selection pressure is different on different base groups in individual proteins. Our results suggest that the majority of protein-coding sequences have evolved in *E. coli* to produce transcription-associated mutations in such a way as to reduce gene knockout, while increasing protein sequence diversity, under stress. Such nonrandom mutagenesis would provide better sets of mutations even before the mutations are exposed to selection. Different selection pressures on *MI* allow each of the protein-coding sequences in the genome to have different evolvability. We discuss the biological benefits of nonrandom *MI* values, how selection shapes *MI* values, and the variation in the selection pressures on *MI* values.

## Results

### Calculation of *MI*

**Figure 1:**
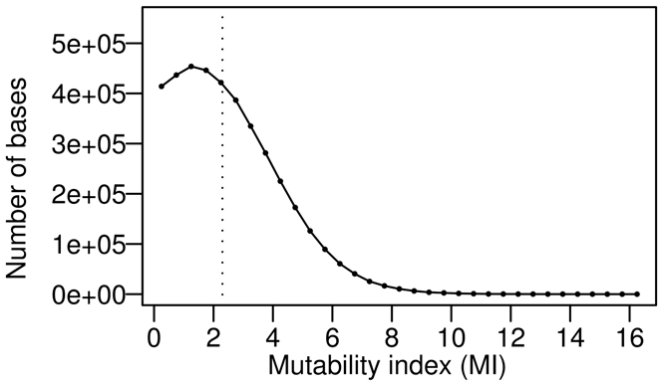
Distribution of the mutability index (*MI*) values of bases located in protein-coding sequences of *E. coli* K12 MG1655. Closed circles indicate the number of bases in each *MI* value range (0.5). The vertical dotted line indicates the mean value (2.54).

Transcription-associated mutability per transcription event should be affected by the local secondary structures of the RNA transcript, on which the functions of noncoding genes depend. Therefore, noncoding genes were excluded from this analysis. *MI* values for the 3,958,572 bases in the 4,132 protein-coding sequences of the *E. coli* K12 MG1655 genome were calculated according to the method described by Wright *et al.* [28], and the negative *MI* values were then converted to zero (see the “Materials and Methods” for details). The mass distribution of the *MI* values was concentrated on smaller *MI* values (0≤*MI*≤2) and exhibited a longer tail at the larger *MI* values (8≤*MI*, skewness = 0.85; Figure 1).

### Evaluation of selection pressures in terms of protein sequence diversity

#### Sequence properties affecting protein sequence diversity

To analyze the selection pressure on *MI* in terms of the generation of protein sequence diversity, or protein evolvability, all the bases in each protein-coding sequence, with the exception of those comprising the start and stop codons, were divided into three groups. The groups were based on the results of the three possible single-base substitutions at that base. The three groups were defined as follows: 1) all three single-base substitutions resulted in silent mutations, which do not change the encoded amino acid (Base_silent_); 2) all three single-base substitutions resulted in missense mutations that changed the encoded amino acid (Base_missense_); and 3) the three single-base substitutions produced two or three types of mutations: silent mutation, missense mutation, or nonsense mutation (Base_other_; Tables 1–2).

**Table 1:** Mutations according to position in the protein-coding sequence.

Bases	Single-base substitution		Insertion or deletion	
	Main results	Main effect	Main result	Effects
**Start codon**	Disruption of start codon	Gene knockout	Frameshift mutations (unless multiples of three bases are inserted or deleted)	Gene knockout or protein truncation, with some functional impairment when it causes a frameshift
**Codons occurring between the start and stop codons**	Only silent mutations (Base_silent_)	Neutral effect		
	Only missense mutations (Base_missense_)	Single amino-acid substitution		
	Mixture of silent, missense, and nonsense mutations (Base_other_)	Various effects		

**Table 2:** Compositions of base types in protein-coding sequences.

Base type	Mean	95% CI
Base_silent_	16.63	16.57–16.69
Base_missense_	56.96	56.90–57.02
Base_other_	26.42	26.32–26.52
Sum	100.0	

#### Generation of control sequences

To analyze the selection pressure on *MI* in terms of the generation of protein-coding sequence diversity, control sequences with the same related features were required. Protein-coding sequences exert most of their effects via their encoded proteins. The compositional ratios of Base_silent_, Base_missense_, and Base_other_ would also affect the potential to generate protein sequence diversity. To comport the same product protein sequence and the same compositional ratios of Base_silent_, Base_missense_, and Base_other_, we generated 100 control sequences per protein-coding sequence by shuffling the positions of the synonymous codons within the protein-coding sequence, except for the start codons and stop codons (Figure S1). The resulting control sequences also had the same GC content as the corresponding protein-coding sequence, which might affect the local secondary structures [3], because they had the same codon usage. The *MI* values of the control sequences were calculated as described above for the protein-coding sequences.

#### Evaluation of the selection pressure on *MI* (S_MI_)

**Figure 2:**
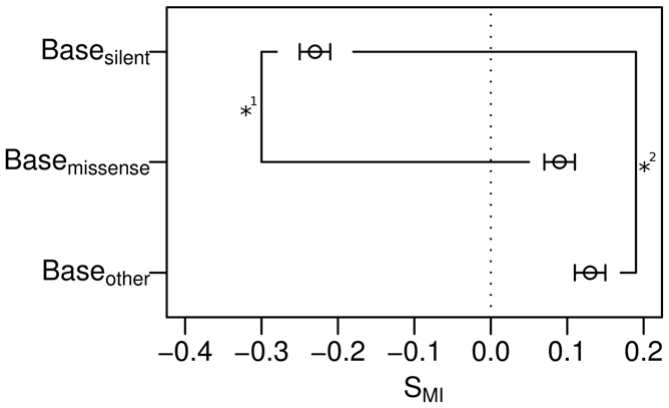
S_MI_ of the base groups according to the mutation types generated by a single-base substitution. The bases were grouped according to the type of mutations caused by single-base substitutions. “Base_silent_” and “Base_missense_” indicate bases where all single-base substitutions at the bases result in silent and missense mutations, respectively. “Base_other_” indicates all bases other than Base_silent_, Base_missense_, and bases that occur in start and stop codons in protein-coding sequences. Circles and error bars indicate the pseudomedian and the 95% confidence interval (CI), respectively, as assessed using the Wilcoxon rank-sum test. The vertical dotted line represents the 0 value for S_MI_. Calculations of the Wilcoxon signed-rank test: *^1^ *P*<1e^−90^; *^2^ *P*<1e^−90^.

The protein-coding sequences and their control sequences have different average potentials to produce transcription-associated mutagenesis per transcription event [3]. By our calculation, the *lacI* gene, for example, has an average *MI* of 2.08 but its control sequences have average *MI* values between 1.78 and 2.42. Transcription-associated mutability is also affected by changes in the transcription level [10]. Therefore, to compare the relative *MI* values of individual bases in each of the protein-coding sequences and the control sequences, standard-normalized *MI* values were introduced into this analysis. The selection pressure on *MI* at Base_silent_, for example, in a protein-coding sequence was evaluated in the following way (Figure S2). All *MI* values were standard normalized within the protein-coding sequence or in each control sequence. All the resulting z-scores of the *MI* values for Base_silent_, for example, were averaged within each protein-coding sequence or within each control sequence. The average z-score for Base_silent_ in the protein-coding sequence was ranked against those in 100 control sequences. The resulting rank values were linearly transformed to “S_MI_” values (−1≤S_MI_≤1), which indicates the selection pressure on *MI* under stress. As a consequence, each protein-coding sequence had one S_MI_ value for Base_silent_. A negative or positive S_MI_ value for Base_silent_ in a protein-coding sequence indicated that the protein-coding sequence had a lower or higher average z-score for the *MI* values at Base_silent_, respectively, than did 50% of the random control sequences. For example, a negative S_MI_ value for a protein-coding sequence at Base_silent_ indicates that the protein-coding sequence has evolved to have lower transcription-associated mutability per transcription event at Base_silent_ under stress. This method of calculating S_MI_ values was also applied to Base_missense_, Base_other_, and all other base groups in the subsequent analyses described in this manuscript. Base_silent_ exhibited lower S_MI_ values than those of Base_missense_ and Base_other_ (Wilcoxon signed-rank test, *P*<1e^−90^ and *P*<1e^−90^, respectively; see “Materials and Methods” for the choice of statistical methods). Base_silent_ and Base_missense_ exhibited distributions of S_MI_ lower and higher than zero, respectively (Wilcoxon rank-sum test, *P*<1e^−100^ and *P*<1e^−100^, respectively; Figure 2). These results suggest that protein-coding sequences have evolved to increase the ratio of missense mutations to silent mutations among transcription-associated mutations under stress. In short, our results suggest that the genome sequence has evolved to increase protein evolvability under stress.

### Selection pressure on *MI* in terms of gene knockout

**Figure 3:**
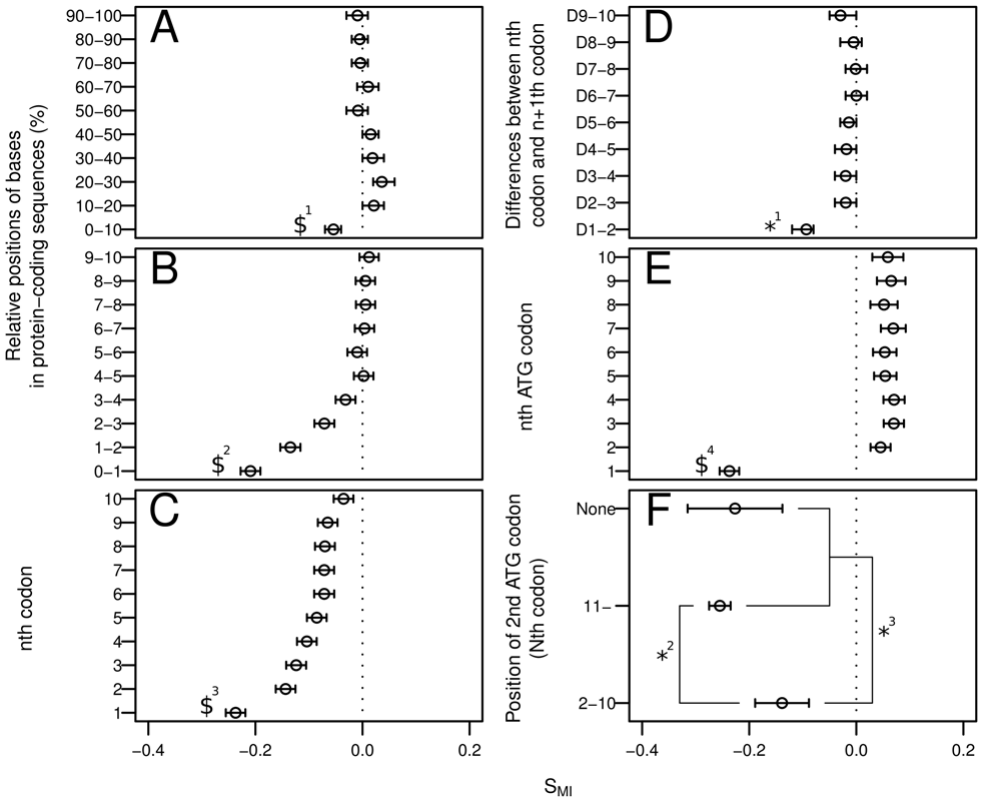
S_MI_ of the base groups according to their relative positions within the protein-coding sequences. (**A**) The relative positions were grouped into deciles from 0 to 100%. (**B**) The relative positions were then grouped into percentiles from 0 to 10%. (**C**) Start codons and their adjacent downstream codons. The 1 on the Y-axis indicates the start codon. (**D**) Difference in the S_MI_ values for the *n* ^th^ codon and the *n*+1^th^ codon. For example, “D1–2” indicates the difference between the S_MI_ values of the first codons (start codons) and those of the second codons. (**E**) Start codons and their following nonstart ATG codons. The 1 on the Y-axis indicates the start codon. (**F**) S_MI_ values for the start codons, according to the positions of the first nonstart ATG codons. $ indicates the largest *P* value among those calculated between a base group marked $ and all the other base groups: $^1^ *P*<0.01; $^2^ *P*<1e^−10^; $^3^ *P*<1e^−20^; $^4^ *P*<1e^−60^; *^1^ *P*<1e^−20^; *^2^ *P*<1e^−4^; *^3^ *P*<1e^−4^ ($^1^, $^3^: Wilcoxon signed-rank test; $^2^, $^4^, *^1^, *^2^, *^3^: Wilcoxon rank-sum test). Circles and error bars indicate the pseudomedian and the 95% CI, respectively, as assessed using the Wilcoxon rank-sum and signed-rank test. The vertical dotted lines represent the 0 value for S_MI_.

Start codons are the sites most likely to be involved in mutation-based gene knockout within protein-coding sequences. To assess the overall distribution of S_MI_ in protein-coding sequences, we grouped the primary positions of protein-coding sequences into deciles, in a 5′ to 3′ direction. Among the 10 base groups, the base group of the first decile showed the lowest distribution of S_MI_ values (Wilcoxon signed-rank test with the division having the second-lowest S_MI_, *P*<0.01; Figure 3A). To analyze this result in greater detail, the first decile was further divided into 1% divisions. Among the resulting 10 groups, the base group of the first percentile showed the lowest distribution of S_MI_ values (Wilcoxon rank-sum test with the division of the second-lowest S_MI_, *P*<1e^−10^; Figure 3B). Start codons occupy the 5′-most positions in protein-coding sequences. This may explain why the base group at the 5′-most position had the lowest S_MI_ value among the groups tested. To confirm this, the bases were grouped for each *n*th codon and the S_MI_ were compared. The start codons displayed the lowest S_MI_ values among the codons located in the 5′ regions of the protein-coding sequences (Wilcoxon signed-rank test with the division of the second-lowest S_MI_, *P*<1e^−20^; Figure 3C). The tendency to form secondary structures is often affected by the primary position on the protein-coding sequence. For instance, bases at the 5′ end are affected by the efficiency of translation initiation and bases at the 3′ end by Rho-independent transcription termination. The base groups of the first four percentiles showed a reduction in S_MI_ values with decreasing percentile (Figure 3B). Therefore, the lowest S_MI_ values observed for the start codons may be caused by this tendency toward low S_MI_ values in the first four percentiles. To exclude this possibility, we compared the results for the subtraction of the S_MI_ values of adjacent codons among the first 10 codons. When the S_MI_ values for the second codons were subtracted from the S_MI_ values for the start codons, the results were less than zero (Wilcoxon rank-sum test, *P*<1e^−20^; Figure 3D). However, the other adjacent codon pairs tested did not yield values significantly less than zero (Wilcoxon rank-sum test, *P*>0.01; Figure 3D). Therefore, the lower S_MI_ values obtained for the start codons are not just a tendency found at the 5′ ends of protein-coding sequences. Start codons exhibit lower S_MI_ values than those of other ATG codons (Wilcoxon rank-sum test, P<1e^−60^; Figure 3E). This result excludes the possibility of a fitness effect exerted by the mutation of ATG codons. To confirm that protein-coding gene knockout influences the effects of the selection pressure on *MI* values, the S_MI_ values for the start codons were compared according to the position of the first nonstart ATG codon. The presence of a closely following nonstart ATG codon at the 5′ end of the protein-coding sequence implies that single-base substitutions at start codons cause the deletion of several *N*-terminal amino acid residues. These *N*-terminal deletions have a lower potential to cause protein-coding gene knockout. The S_MI_ values of start codons were higher in the presence of nonstart ATG codons in the first 10 codons than in the presence of nonstart ATG codons after the first 10 codons (Wilcoxon rank-sum test, *P*<1e^−4^; Figure 3F) and in the absence of nonstart ATG codons within the first 10 codons (Wilcoxon rank-sum test, *P*<1e^−4^; Figure 3F). These results demonstrate that selection acts to reduce the *MI* values of start codons when start codon mutations cause gene knockout. These results suggest that protein-coding sequences have evolved to reduce the proportion of gene knockouts by transcription-associated mutations under stress.

### Correlation between before and after single-base substitutions

A single-base substitution in ssDNA often changes the most stable secondary structure of the ssDNA. Therefore, a single-base substitution of a base may change the *MI* value of the base itself and of its 58 neighboring bases (29 bases upstream and 29 bases downstream; Figure S3). The altered *MI* value would affect the positive and negative selection that shapes the nonrandom *MI*. To examine how nonrandom *MI* distributions are shaped, it is important to assess the mutational effect of a specific base on its own *MI* value and on the *MI* values of neighboring bases. Therefore, we repeated 10,000 random single-base substitutions 100 times and analyzed the correlation values of the *MI* values for the mutated bases themselves and for their neighboring bases before and after single-base substitutions (see “Materials and Methods”). The *MI* values showed significant positive correlations at both the mutated bases (R^2^ = 0.29, *P*<1e^−100^) and the neighboring bases (R^2^ = 0.79, *P*<1e^−100^). The bases with high *MI* values tended to retain similar *MI* values after single-base substitutions (Figure S4). These results demonstrate that the *MI* values of bases are usually subject to larger variation when mutations occur at the base itself than when mutations occur at neighboring bases, and that bases with high *MI* values are more tolerant of variation in their *MI* values after single-base substitutions at those bases than are bases with low *MI* values.

## Discussion

### Biological benefits of the nonrandom distribution of *MI* values under stress

In this study, we have demonstrated that selection acts on protein-coding sequences to lower the *MI* values at Base_silent_ and to increase the *MI* values at Base_missense_ and Base_other_ under stress (Figure 2). Under stress, protein sequence diversity is preferred and some of this diversity is beneficial. Most beneficial mutations are missense mutations rather than indels [31]. Missense mutations can occur at Base_missense_ and Base_other_ but not at Base_silent_. However, some missense mutations can have detrimental effects. Therefore, under stress, high *MI* at Base_missense_ and Base_other_ and low *MI* at Base_silent_ can increase the chance of beneficial mutations at the cost of increasing detrimental mutations. The proportion of detrimental mutations among indels is larger than the proportion of detrimental mutations among single-base substitutions. Therefore, the proportion of detrimental mutations among indels is larger than the proportion of detrimental mutations among indels plus single-base substitutions. A protein sequence can be mutated only by indels at Base_silent_ and by both indels and single-base substitutions at Base_missense_ and Base_other_. Therefore, the proportion of detrimental mutations among mutations that result in a change in the protein sequence is larger at Base_silent_ than at Base_missense_ and Base_other_.

These results imply that protein-coding sequences have evolved to reduce the proportion of detrimental mutations produced while increasing protein sequence diversity under stress. Supporting this proposition, the *MI* values at start codons are low when mutations at start codons are likely to cause gene knockouts (Figure 3). Gene knockouts often result in detrimental mutations, for example in essential genes, and often exert disadvantageous effects. In prokaryotes such as *E. coli*, selective pressure is exerted on the genome length, so they have very compact genomes [32]. This implies that most preexisting genes recently contributed to the fitness of the cell before they were lost in response to the selection pressure on genome size. Consequently, our data suggest that protein-coding sequences have evolved to control the transcription-associated mutability per transcription event to increase protein evolvability and to reduce the proportion of detrimental mutations produced under stress.

### Selection type that shapes nonrandom *MI* values within protein-coding sequences

The reduction of *MI* values at Base_silent_ and start codons can be achieved by purifying selection. Detrimental mutations are not passed on to the descendant cells. We have demonstrated that mutations alter the *MI* values of neighboring bases as well as that of the mutated base itself. Therefore, the different *MI* values of start codons and Base_silent_ can be caused by mutations at neighboring bases. *E. coli* cells and their descendants that have start codons and Base_silent_ with high *MI* values would frequently suffer detrimental mutations because mutations occur frequently at these sites. Increases in the *MI* values at Base_missense_ can be achieved by positive selection. Positive selection implies that the mutated bases are inherited by the descendant cells and become dominant. The new bases at these sites will have different *MI* values. However, as we have demonstrated, new *MI* values correlate positively with the old *MI* values at the mutated sites. In particular, bases with high *MI* values tend to retain these high *MI* values after single-base substitutions (Figure S4). Therefore, high *MI* values at Base_missense_ are frequently retained, although some degree of fluctuation cannot be avoided. The distribution of S_MI_ values at Base_other_ was closer to that at Base_missense_ than to that at Base_silent_ (Figure 2). Single-base substitutions at Base_other_ are made up of two or more types of mutations: silent, missense, and nonsense mutations. The compositions of silent mutations, missense mutations, and nonsense mutations at Base_other_ were 26%, 60%, and 14%, respectively, throughout all protein-coding sequences. Because the proportion of missense mutations is highest at Base_other_, the *MI* values at Base_other_ will be influenced by selection in a similar way to those at Base_missense_ rather than to those at Base_silent_.

### Variation in the selection pressure on *MI* values

Transcription-associated mutations increase under stress and in highly transcribed regions. Individual protein-coding sequences are repressed or derepressed under different stresses and have different transcription levels under specific stresses. Therefore, each protein-coding sequence has a different S_MI_ value for each of its base groups (Table 3). This result suggests that the protein products of individual protein-coding sequences have different evolvability. Interestingly, about one third of protein-coding sequences have S_MI_ values that are larger than zero at Base_silent_ (Table 3). This suggests that about one third of protein-coding sequences have evolved to reduce protein sequence diversity under stress. Reduced protein evolvability is beneficial under favorable conditions. Therefore, the high S_MI_ values at Base_silent_ and the low S_MI_ values at Base_missense_ and Base_other_ might be attributable to the activity of transcription-associated mutagenesis under favorable conditions. However, the development of an index that predicts transcription-associated mutability per transcription event under favorable conditions and its subsequent analysis will be necessary to determine the mechanism underlying this phenomenon.

**Table 3:** Distributions of S_MI_ values.

	−1.0≤S_MI_<−0.5	−0.5≤S_MI_<0	0≤S_MI_<0.5	0.5≤S_MI_≤1	Sum
Base_missense_	21.25%	21.03%	25.46%	32.26%	100.0%
Base_other_	19.53%	21.93%	24.25%	34.29%	100.0%
Base_silent_	38.53%	25.62%	20.57%	15.27%	100.0%

### Conclusions

In this study, we evaluated the selection pressure on *MI*, which represents the relative potential for transcription-associated mutagenesis per transcription event under stress. Our results suggest that the majority of protein-coding sequences have evolved to increase protein sequence diversity by controlling transcription-associated mutagenesis under stress and that transcription-associated mutagenesis produces protein sequence diversity more effectively than does random mutagenesis. Therefore, transcription-associated mutagenesis will confer faster protein evolvability under stress, and will improve the chance of survival of *E. coli*.

## Materials and Methods

### Sequence data

The sequence of *E. coli* K12 MG1655 was downloaded from the NCBI ftp site (ftp://ftp.ncbi.nih.gov/genomes/Bacteria/Escherichia_coli_K12). The sequences of the 4,132 *E. coli* protein-coding genes were used in this analysis. For each protein-coding sequence, 100 control sequences were generated by synonymous codon shuffling; the codon position for any given amino acid was shuffled, whereas the positions of the start and stop codons were maintained (see Figure S1). This step was repeated for each of the 20 amino acids. Random shuffling was performed using the “random” module of the standard library of the Python program, version 2.6 (http://www.python.org).

### Sequence features

#### Relative base positions in protein-coding sequences

The base pairs in a protein-coding sequence with a length of *n* base pairs were numbered from 1 to *n*. For the *k* ^th^ base, the length ratio was defined as ([*k*–1]/[*n*–1])×100. For example, the first base of the start codon and the last base of the stop codon were assigned positions 0 and 100, respectively.

#### *n* ^th^ codon

In a protein-coding sequence that produces a protein of *n* amino acids, the codons were numbered from 1 to *n*+1. For example, the start and stop codons were assigned positions 1 and *n*+1, respectively.

#### *n* ^th^ ATG codon

In a protein-coding sequence, the start codon was assigned position 1, even when it was not an ATG. The subsequent *k* ATG codons were sequentially assigned positions from 2 to *k*+1.

### Selection of a method to predict transcription-associated mutability per transcription event

Two methods have been developed to predict transcription-associated mutagenesis potentials. The first is the *MI* of Wright *et al.*, which focuses on transcription-associated mutations and was validated using *in vivo* mutation data from reversion assays. Because the purpose of this study was to investigate the selection pressure on transcription-associated mutability per transcription event under stress, Wright et al.'s *MI* was adequate. Hoede *et al.* [3] developed another index, the transcription-driven mutability index (*TDMI*). To validate these indices, these authors used conservation data taken from the alignment of orthologous gene sequences among *E. coli* strains. The mutations that generated these variable sites might be originated under stress, under favorable conditions, or under a mixture of both types of conditions. The variable sites in the sequence alignments were the results of mutations in the ancestral sequences. Therefore, the *TDMI* values for the ancestral sequences should have been compared with the conservation data in the validation process. However, the *TDMI* values were calculated from the sequence of an extant *E. coli* strain. Therefore, *TDMI* was not adequate for the present analysis.

### Calculation of *MI* values

We initially calculated the *MI* values according to the method described by Wright *et al.* [28] using the hybrid-ss-min program of the UNAFold package [33], which is the local program from the DINAMelt web server, version 3.6 [34], used by Wright *et al.* The calculation of *MI* with an average ssDNA length of 30 nt was described here. The protein-coding sequences were extended to include the 29-nt (that is [30]–[1]-nt) upstream and downstream sequences. Subsequences were then generated by sliding a window of 30 nt along the resulting extended sequence. Consequently, each individual base belonged to 30 subsequences. All subsequences were then folded using the hybrid-ss-min program [33] and the ΔG values and paired/unpaired status of the most stable folds of each given subsequence were determined. The *MI* value for each base was calculated using the equation proposed by Wright *et al.* [28]: (number of folds in which the base is unpaired)/30×(highest –ΔG of all 30 folds in which the base is unpaired). A small proportion of *MI* values (0.4%) was smaller than zero when the first factor, ‘number of folds in which the base is unpaired’, was greater than zero but the second factor, ‘highest –ΔG of all *n* folds in which the base is unpaired’, was smaller than zero. However, when the ‘number of folds in which the base is unpaired’ is zero, the *MI* value becomes zero. This contradicts the assumption that bases that remain unpaired for a longer time have higher *MI* values. Stable folds are not formed when the –ΔG of the most stable fold is less than zero. Therefore, these values were converted to zero.

### Evaluation of the effect of selection on *MI* (S_MI_)

For protein-coding sequences and their control sequences, the *MI* value of each base was standard normalized within the protein-coding sequence. To compare the transcription-associated mutability per transcription event among the base groups, the standard-normalized *MI* values for each base group were averaged for the protein-coding sequence and for each of the control sequences. The rank of a gene sequence at a given base group was calculated from these averaged values against the corresponding values of 100 control sequences. The rank was then transformed to S_MI_ values using the equation (2×rank – 102)/100. As a result, each protein-coding sequence had a value between −1 and 1 (−1≤S_MI_≤1) for each base group.

### Correlation of *MI* values before and after mutation

Ten thousand single bases selected randomly from protein-coding sequences were randomly mutated to one of the three other possible bases. The *MI* values of the base targeted by the mutation and of the 29 bases located upstream and downstream from this position were calculated before and after the mutation. The neighboring bases that were not within the protein-coding sequences were excluded from the calculation of the correlation. The *MI* values of the same bases before and after mutation were used to calculate Pearson's correlation values. These steps were repeated 100 times. The mean Pearson's correlation values were used for this study.

### Selection of statistical methods

Wilcoxon signed-rank test and Wilcoxon rank-sum test are nonparametric statistical methods and are alternatives to paired Student's *t* test and Student's *t* test, respectively. Nonparametric statistical methods are robust and can be used even when a normal distribution cannot be assumed. To compare the difference of groups, the Wilcoxon signed-rank test and Wilcoxon rank-sum test were used. We used paired comparisons (and hence the Wilcoxon signed-rank test) wherever possible. For example, in Figure 3C, the Wilcoxon signed-rank test was used because every protein-coding sequence had more than 10 codons, so a paired comparison of the start codons and *i*th codons (2≤*i*≤10) inside each of the protein-coding sequences was possible. Conversely, in Figure 3E, the Wilcoxon rank-sum test was used because some protein-coding sequences did not have 10 ATG codons and paired comparisons were not possible in some protein-coding sequences.

All statistical analyses were performed using the R statistical package [35]. All rankings were made in increasing order.

## Supporting Information

Figure S1 Schematic representation of synonymous codon shuffling. (A) An imaginary sequence encoding six amino acids. The start codon and stop codon were excluded from shuffling. The arrows indicate random shuffling. The positions of synonymous codons (encoding the same amino acid) were shuffled randomly. This shuffling was repeated for each of the 20 amino acids. In the example sequence, there is one codon for serine, so it was self-shuffled. (B) All the control sequences generated from the sequence in (A). The control sequences can include the protein-coding sequence if the synonymous-codon-shuffled sequences happen to include the same sequence as the protein-coding sequence.
(0.01 MB PDF)

Figure S2 Example of calculating S_MI_ from arbitrary *MI* values. (A) *MI* values for an imaginary protein-coding sequence and its control sequences. The control sequences were generated with synonymous codon shuffling. The z-score values for *MI* were arbitrarily assigned for the demonstration. Base groups START, O, M, S, and STOP represent the start codon, Base_other_, Base_missense_, Base_silent_, and the stop codon, respectively. (B) The average z-scores for *MI* for the individual base groups of individual sequences. For example, Base_silent_ of the protein-coding sequence has z-scores for the *MI* values of − 2.01, 2.95, and 0.42 in (A), so its average value (0.45) is written as the corresponding value in (B). (C) The z-score average for *MI* of the protein-coding sequence was ranked among the corresponding values for the control sequences. The rank values (1≤rank≤N+1) obtained were linearly transformed to S_MI_ (− 1≤S_MI_≤1) using the equation (2×rank − 2 − N)/N, where N is the number of control sequences (which is four in this example and 100 in the main text). In this example, four control sequences were used for clarity and the values were rounded to the nearest two decimal places, although we used 100 control sequences and calculated them to 15 decimal places in the actual calculation in the main text.
(0.34 MB PDF)

Figure S3 An example of the effect of a single-base substitution on secondary structure. (A) A single-base substitution can affect the most stable secondary structures of all ssDNA sequences that contain the mutated site. Each underlined sequence is 30 nt. (B) The sequence underlined by third line in (A). (C) The resulting sequence of G-to-C single-base substitution at the base marked with a star in the sequence shown in (B). The secondary structures of (B) and (C) indicate the most stable secondary structures of these sequences. In this example, the G-to-C mutation changes the most stable secondary structure and hence the paired/unpaired state and the −ΔG value of the bases, which determine their *MI* values.
(0.54 MB TIF)

Figure S4 Correlation of *MI* values before and after single-base substitutions. The correlations of the *MI* values before and after mutation were plotted. The *MI* values were divided into 10 groups according to the rank of each before and after a single-base substitution. The diameter of each circle is proportional to the number in each division.
(0.57 MB TIF)
