# Supplementary material for: Transcription-Associated Mutagenesis Increases Protein Sequence Diversity More Effectively than Does Random Mutagenesis in Escherichia coli
Source: PLoS One. 2010 May 10;5(5):e10567. doi: 10.1371/journal.pone.0010567 (PMC2866735; doi:10.1371/journal.pone.0010567)
Supplement: Figure S1 — Schematic representation of synonymous codon shuffling. (A) An imaginary sequence encoding six amino acids. The start codon and stop codon were excluded from shuffling. The arrows indicate random shuffling. The positions of synonymous codons (encoding the same amino acid) were shuffled randomly. This shuffling was repeated for each of the 20 amino acids. In the example sequence, there is one codon for serine, so it was self-shuffled. (B) All the control sequences generated from the sequence in (A). The control sequences can include the protein-coding sequence if the synonymous-codon-shuffled sequences happen to include the same sequence as the protein-coding sequence. (0.01 MB PDF) [file pone.0010567.s001.pdf]

A

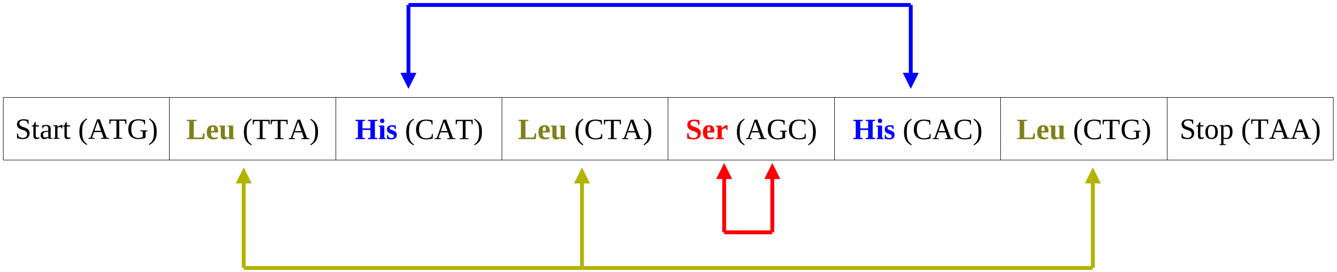

B

ATGTTACATCTAAGCCACCTGTAA
ATGCTGCACCTAAGCCATTTATAA
ATGCTACATCTGAGCCACTTATAA
ATGCTACACCTGAGCCATTTATAA
ATGTTACATCTGAGCCACCTATAA
ATGCTGCACTTAAGCCATCTATAA
ATGCTACATTTAAGCCACCTGTAA
ATGCTACACTTAAGCCATCTGTAA
ATGTTACACCTAAGCCATCTGTAA
ATGCTGCATTTAAGCCACCTATAA
ATGTTACACCTGAGCCATCTATAA
ATGCTGCATCTAAGCCACTTATAA
