# Supplementary material for: Transcription-Associated Mutagenesis Increases Protein Sequence Diversity More Effectively than Does Random Mutagenesis in Escherichia coli
Source: PLoS One. 2010 May 10;5(5):e10567. doi: 10.1371/journal.pone.0010567 (PMC2866735; doi:10.1371/journal.pone.0010567)

A

Amino acid sequence		M			R			R			R			R			*		
Protein-coding sequence	Sequence	A	T	G	A	G	G	C	G	A	C	G	C	C	G	G	T	A	A
	Z-score of MI	0.37	-0.01	0.25	1.81	-0.27	-0.63	-0.02	0.52	-2.01	0.34	-1.25	2.95	0.58	-0.52	0.42	-0.41	0.55	1.05
	Base group	START			O	M	O	O	M	S	M	M	S	O	M	S	STOP		
Control #1	Sequence	A	T	G	C	G	A	C	G	G	C	G	C	A	G	G	T	A	A
	Z-score of MI	0.41	0.44	-0.64	0.04	1.80	0.16	-0.43	0.95	-0.02	0.93	-0.43	0.26	-1.27	-1.84	0.00	0.96	-0.69	0.23
	Base group	START			O	M	S	O	M	S	M	M	S	O	M	O	STOP		
Control #2	Sequence	A	T	G	C	G	G	C	G	C	A	G	G	C	G	A	T	A	A
	Z-score of MI	-0.59	0.52	-1.72	-0.04	-0.35	-0.27	0.21	-1.16	-1.30	1.32	1.53	1.06	2.12	-0.16	-1.09	0.59	-0.30	-0.50
	Base group	START			O	M	S	M	M	S	O	M	O	O	M	S	STOP		
Control #3	Sequence	A	T	G	C	G	G	A	G	G	C	G	C	C	G	A	T	A	A
	Z-score of MI	0.41	0.90	-0.45	-0.52	-1.02	-2.35	-0.93	0.59	-0.57	-0.26	0.66	0.73	1.21	0.20	-2.53	0.39	0.56	-2.21
	Base group	START			O	M	S	O	M	O	M	M	S	O	M	S	STOP		
Control #4	Sequence	A	T	G	A	G	G	C	G	A	C	G	C	C	G	G	T	A	A
	Z-score of MI	-0.64	-0.32	-0.29	-2.41	0.22	0.62	-2.20	2.28	-1.83	-0.17	0.29	-1.11	1.37	-1.44	-0.26	-1.05	-1.02	-0.41
	Base group	START			O	M	O	O	M	S	M	M	S	O	M	S	STOP		

B

	MI average				
	Start codon	$Base_{silent}$	$Base_{missense}$	$Base_{other}$	Stop codon
Protein-coding sequence	0.20	0.45	-0.24	0.44	0.40
Control #1	0.07	0.13	0.28	-0.42	0.17
Control #2	-0.60	-0.89	0.01	1.12	-0.07
Control #3	0.29	-1.38	0.03	-0.20	-0.42
Control #4	-0.42	-1.07	0.24	-0.66	-0.83
Rank of protein-coding sequence among all control sequences	4	5	1	4	5

C

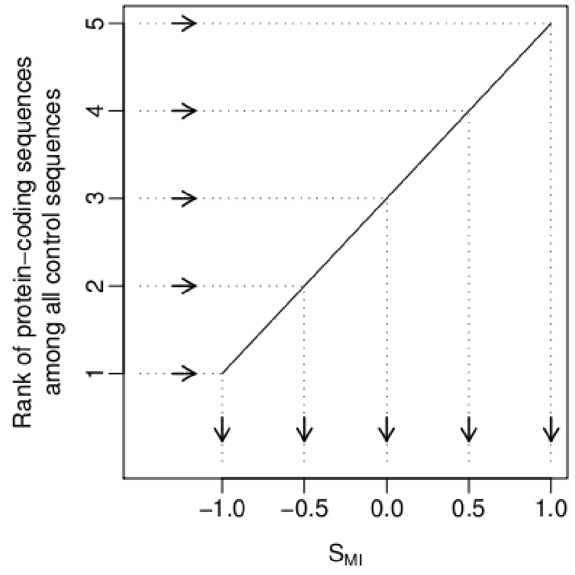

Supplement: Figure S2 — Example of calculating SMI from arbitrary MI values. (A) MI values for an imaginary protein-coding sequence and its control sequences. The control sequences were generated with synonymous codon shuffling. The z-score values for MI were arbitrarily assigned for the demonstration. Base groups START, O, M, S, and STOP represent the start codon, Baseother, Basemissense, Basesilent, and the stop codon, respectively. (B) The average z-scores for MI for the individual base groups of individual sequences. For example, Basesilent of the protein-coding sequence has z-scores for the MI values of − 2.01, 2.95, and 0.42 in (A), so its average value (0.45) is written as the corresponding value in (B). (C) The z-score average for MI of the protein-coding sequence was ranked among the corresponding values for the control sequences. The rank values (1≤rank≤N+1) obtained were linearly transformed to SMI (− 1≤SMI≤1) using the equation (2×rank − 2 − N)/N, where N is the number of control sequences (which is four in this example and 100 in the main text). In this example, four control sequences were used for clarity and the values were rounded to the nearest two decimal places, although we used 100 control sequences and calculated them to 15 decimal places in the actual calculation in the main text. (0.34 MB PDF) [file pone.0010567.s002.pdf]
