# Supplementary figures and images for: Transcription-Associated Mutagenesis Increases Protein Sequence Diversity More Effectively than Does Random Mutagenesis in Escherichia coli
Source: PLoS One. 2010 May 10;5(5):e10567. doi: 10.1371/journal.pone.0010567 (PMC2866735; doi:10.1371/journal.pone.0010567)

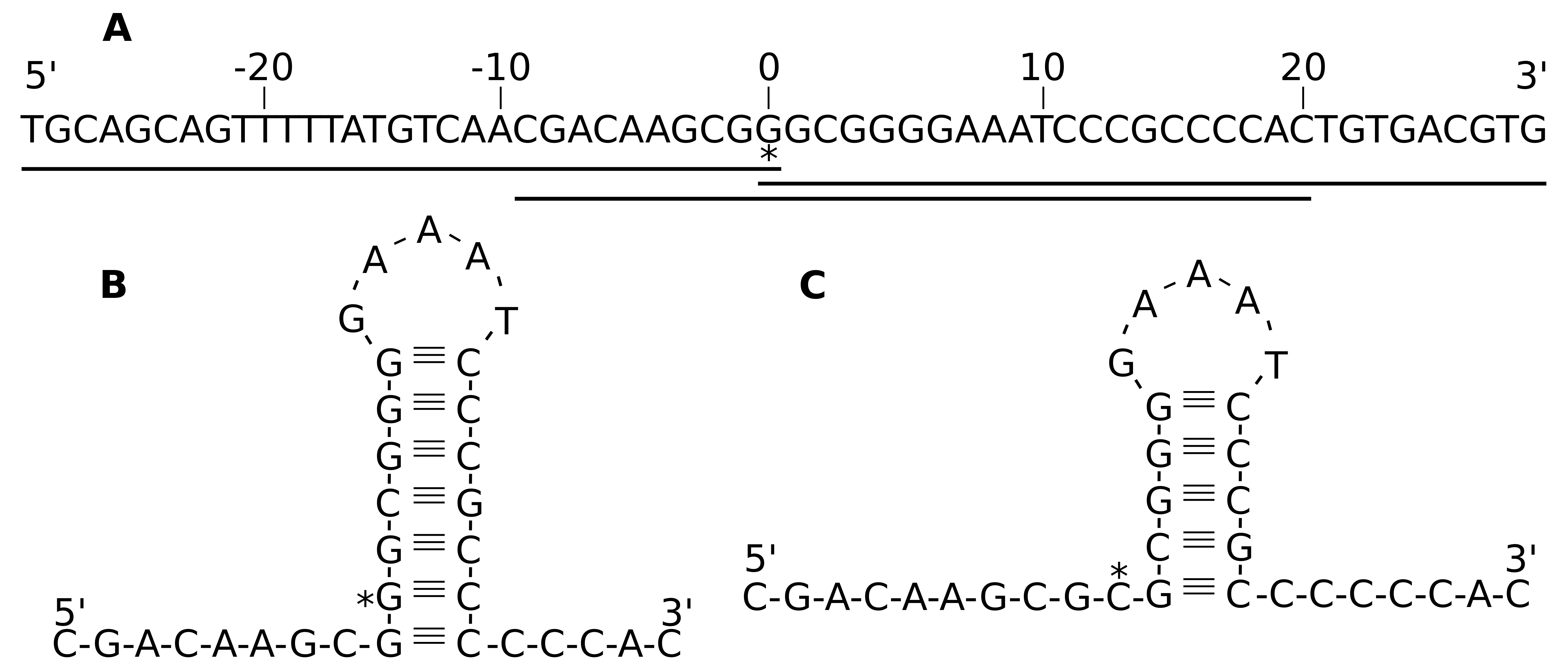

Supplement: Figure S3 — An example of the effect of a single-base substitution on secondary structure. (A) A single-base substitution can affect the most stable secondary structures of all ssDNA sequences that contain the mutated site. Each underlined sequence is 30 nt. (B) The sequence underlined by third line in (A). (C) The resulting sequence of G-to-C single-base substitution at the base marked with a star in the sequence shown in (B). The secondary structures of (B) and (C) indicate the most stable secondary structures of these sequences. In this example, the G-to-C mutation changes the most stable secondary structure and hence the paired/unpaired state and the −ΔG value of the bases, which determine their MI values. (0.54 MB TIF) [file pone.0010567.s003.tif]

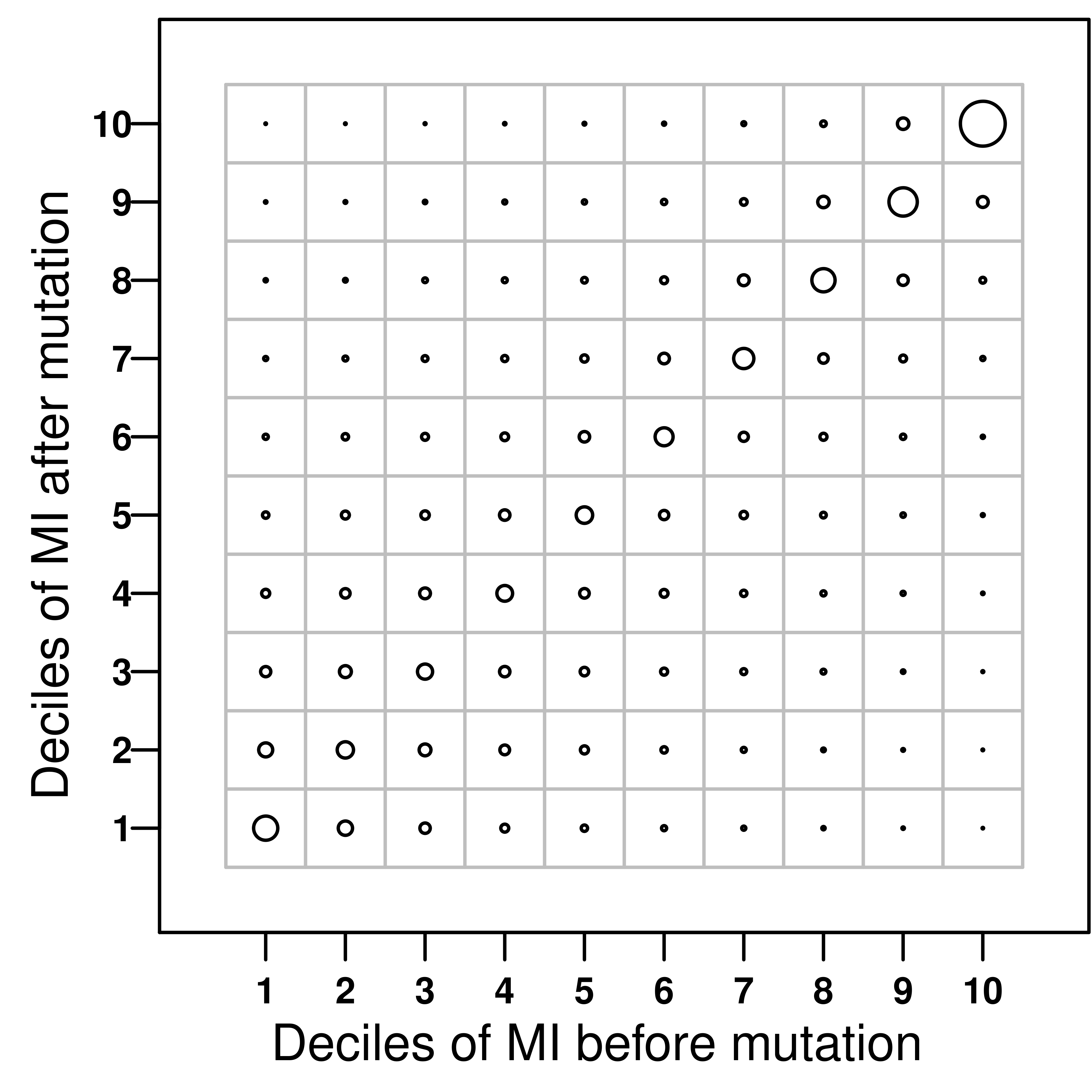

Supplement: Figure S4 — Correlation of MI values before and after single-base substitutions. The correlations of the MI values before and after mutation were plotted. The MI values were divided into 10 groups according to the rank of each before and after a single-base substitution. The diameter of each circle is proportional to the number in each division. (0.57 MB TIF) [file pone.0010567.s004.tif]
